# The *in Vitro* Antigenicity of *Plasmodium vivax* Rhoptry Neck Protein 2 (*Pv*RON2) B- and T-Epitopes Selected by HLA-DRB1 Binding Profile

**DOI:** 10.3389/fcimb.2018.00156

**Published:** 2018-05-15

**Authors:** Carolina López, Yoelis Yepes-Pérez, Diana Díaz-Arévalo, Manuel E. Patarroyo, Manuel A. Patarroyo

**Affiliations:** ^1^Molecular Biology and Immunology Department, Fundación Instituto de Inmunología de Colombia, Bogotá, Colombia; ^2^PhD Program in Biomedical and Biological Sciences, Universidad del Rosario, Bogotá, Colombia; ^3^MSc Program in Microbiology, Universidad Nacional de Colombia, Bogotá, Colombia; ^4^Faculty of Agricultural Sciences, Universidad de Ciencias Aplicadas y Ambientales, Bogotá, Colombia; ^5^School of Medicine, Universidad Nacional de Colombia, Bogotá, Colombia; ^6^Basic Sciences Department, School of Medicine and Health Sciences, Universidad del Rosario, Bogotá, Colombia

**Keywords:** *Plasmodium vivax*, *Pv*RON2, HLA-DRB1 typing, antigenicity, synthetic peptide, epitope, cellular and humoral response

## Abstract

Malaria caused by *Plasmodium vivax* is a neglected disease which is responsible for the highest morbidity in both Americas and Asia. Despite continuous public health efforts to prevent malarial infection, an effective antimalarial vaccine is still urgently needed. *P. vivax* vaccine development involves analyzing naturally-infected patients' immune response to the specific proteins involved in red blood cell invasion. The *P. vivax* rhoptry neck protein 2 (*Pv*RON2) is a highly conserved protein which is expressed in late schizont rhoptries; it interacts directly with AMA-1 and might be involved in moving-junction formation. Bioinformatics approaches were used here to select B- and T-cell epitopes. Eleven high-affinity binding peptides were selected using the NetMHCIIpan-3.0 *in silico* prediction tool; their *in vitro* binding to HLA-DRB1^*^0401, HLA-DRB1^*^0701, HLA-DRB1^*^1101 or HLA-DRB1^*^1302 was experimentally assessed. Four peptides (39152 (HLA-DRB1^*^04 and 11), 39047 (HLA-DRB1^*^07), 39154 (HLADRB1^*^13) and universal peptide 39153) evoked a naturally-acquired T-cell immune response in *P. vivax*-exposed individuals from two endemic areas in Colombia. All four peptides had an SI greater than 2 in proliferation assays; however, only peptides 39154 and 39153 had significant differences compared to the control group. Peptide 39047 was able to significantly stimulate TNF and IL-10 production while 39154 stimulated TNF production. Allele-specific peptides (but not the universal one) were able to stimulate IL-6 production; however, none induced IFN-γ production. The Bepipred 1.0 tool was used for selecting four B-cell epitopes *in silico* regarding humoral response. Peptide 39041 was the only one recognized by *P. vivax*-exposed individuals' sera and had significant differences concerning IgG subclasses; an IgG2 > IgG4 profile was observed for this peptide, agreeing with a protection-inducing role against *P. falciparum* and *P. vivax* as previously described for antigens such as RESA and MSP2. The bioinformatics results and *in vitro* evaluation reported here highlighted two T-cell epitopes (39047 and 39154) being recognized by memory cells and a B-cell epitope (39041) identified by *P. vivax*-exposed individuals' sera which could be used as potential candidates when designing a subunit-based vaccine.

## Background

Malaria is one of the most important public health problems in tropical and subtropical regions worldwide. Nearly 3.3 billion people globally are at risk of contracting the disease; 214 million new cases appeared and 438,000 deaths occurred in 2015. Malaria is caused by parasites from the phylum Apicomplexa, genus *Plasmodium. Plasmodium vivax* is the second most prevalent known species and has the greatest geographical distribution as it can develop in its vector at lower temperatures and survive at higher altitudes. It also has a latent form known as hypnozoite; this remains in the hepatocytes, enabling parasite survival in a host for a long time (Mueller et al., 2009; Guerra et al., 2010; WHO, 2016).

Although malarial cases in Latin America decreased during the last decade, a rise in cases reported from Venezuela and Colombia has been reported in 2015 and 2016 (PAHO/WHO, 2017), Colombia being listed as the fourth regarding incidence during 2015 (i.e., 10% of malarial events) (WHO, 2016). A passive surveillance study of malarial transmission in Colombia between 2011 and 2013 showed that 50.7% of cases were caused by *P. vivax*, 48.9% by *P. falciparum* and 0.4% mixed infection (Arévalo-Herrera et al., 2015). A severe malaria study on Colombia's Pacific coast showed that *P. vivax* induced acute anemia in children and *P. falciparum* patients had high renal and hepatic damage rates (Arévalo-Herrera et al., 2017).

Since *P. vivax* has wide-scale global distribution, some strategies used to combat malaria involve using insecticide-impregnated mosquito nets and drugs such as sulphadoxine-pyrimethamine, artemisinin, and chloroquine (WHO, 2016); despite such efforts, vector insecticide resistance and parasite resistance to anti-malarial drug has increased during recent years (Rieckmann et al., 1989; Fairhurst and Dondorp, 2016). Administering anti-malarial drugs, together with developing an effective antimalarial vaccine, is considered a relevant control strategy for preventing and eradicating malaria (WHO, 2016).

More than 50 proteins have been described to date as being involved in malarial parasite's red blood cell (RBC) invasion; most have been identified at molecular level and characterized immunologically in *P. falciparum* (Bozdech et al., 2003; Cowman and Crabb, 2006). Conversely, studying *P. vivax* proteins involved in host invasion has been difficult, mainly due to technical restrictions such as the lack of a continuous *in vitro* parasite culture, leading to inadequate study of parasite biology (Udomsangpetch et al., 2008; Mueller et al., 2009).

Parasites from the phylum *Apicomplexa* have specialized organelles such as rhoptries which contain a large amount of proteins involved in host cell invasion (Counihan et al., 2013). Six *P. vivax* rhoptry neck proteins have been identified to date: *Pv*34, *Pv*RON1, *Pv*RON2, *Pv*RON4, *Pv*RALP and *Pv*RON5 (Mongui et al., 2009; Arévalo-Pinzón et al., 2011, 2013, 2015; Moreno-Perez et al., 2011; Cheng et al., 2015). They have been described as possible targets for blocking *P. vivax* invasion of RBC (Mongui et al., 2009).

*P. vivax* rhoptry neck protein 2 (*Pv*RON2) is 2,204 amino acids (aa) long and is expressed in late schizont rhoptries (Arévalo-Pinzón et al., 2011). It is a highly conserved protein which is secreted by specialized organelles and forms part of the complex of proteins called RONs. This protein, like its orthologs in *T. gondii* (*Tg*RON2) and *P. falciparum* (*Pf* RON2) interacts directly with the AMA-1 protein. The RON complex is involved in forming the moving junction (MJ) (electro dense ring-shaped structure) which allows parasite entry to a host cell (Aikawa et al., 1978; Lamarque et al., 2011). RON2's crucial role during merozoite (Mrz) invasion of erythrocytes, moving junction (MJ) formation and subsequent parasitophorous vacuole (PV) formation (Cao et al., 2009; Collins et al., 2009; Srinivasan et al., 2011) makes it a good vaccine candidate. Moreover, Srinivasan *et al*. have shown that vaccination with the *Pf* AMA1-RON2L complex induce protection in *Aotus* monkeys, mediated by high neutralizing antibody titers that prevent the invasion of RBC (Srinivasan et al., 2017).

The development of bioinformatics tools during the last few decades has enabled predicting vaccine candidates based on peptide binding affinity for major histocompatibility complex (MHC) class I or class II (Sturniolo et al., 1999; Nielsen and Lund, 2009; Wang et al., 2010; Zhang et al., 2012; Andreatta et al., 2015). The immune system's function is to recognize and differentiate between self and non-self-antigens so as to trigger cellular and/or humoral immune responses. MHC class II proteins (HLA-II in humans) are expressed on antigen presenting cells' (APC) surface (i.e., macrophages, dendritic cells and B-lymphocytes). These recognize extracellular antigens and can bind 13- to 18-aa-long peptides. One of the main difficulties in designing a vaccine is the high HLA polymorphism, especially from HLA-DRB1, this being the most polymorphic locus. Antigen binding capability varies from one allele to another, increasing or reducing affinity and driving immune responses. Selecting peptides as good vaccine candidates relies on their ability to be recognized by HLA-DRB1 alleles to ensure a protection-inducing immune response (Stern and Calvo-Calle, 2009). T-cells can trigger stronger immune responses after their APC recognition, depending on the peptides bound to MHC receptors (Blum et al., 2013).

Antigen-antibody interaction plays an essential role in humoral immune responses against pathogens. Bioinformatics tools are extremely useful for identifying antigenic determinants or B-cell epitopes when designing vaccines (Bergmann-Leitner et al., 2013; Panda and Mahapatra, 2017). Predicting linear B-cell epitopes (contiguous aa in a protein sequence) is based on several methods for determining aa physicochemical properties, such as solvent accessibility, hydrophilicity and flexibility (El-Manzalawy et al., 2017; Solihah et al., 2017).

This paper describes naturally-acquired T-cell and antibody immune responses to *Pv*RON2 in *P. vivax*-exposed individuals from two of Colombia's endemic areas (Córdoba and Chocó), in the search for vaccine candidates. Eleven high-affinity epitopes were selected by NetMHCIIpan-3.0 (Andreatta et al., 2015) *in silico* prediction and their *in vitro* binding to at least one of HLA-DRB1^*^0401, HLA-DRB1^*^0701, HLA-DRB1^*^1101 and HLA-DRB1^*^1302 was assessed by competition assays. The Bepripred 1.0 tool was used for selecting four B-cell epitopes *in silico*. A good immune response was observed against two T-cell and one B-cell epitopes; further studies aimed at testing these peptides as components of a subunit vaccine against *P. vivax* are thus recommended.

## Materials and methods

### *In silico* B-cell and T-cell epitope high binding prediction

The *Pv*RON2 aa sequence (PlasmoDB database code: PVX_117880) was used for predicting T-cell epitopes having high binding affinity for the HLA-DRB1 alleles most frequently occurring in endemic areas worldwide (HLA-DRB1^*^0401, HLA-DRB1^*^0701, HLA-DRB1^*^1101, and HLA-DRB1^*^1302) (Marsh et al., 1999). NetMHCIIpan-3.0 (Andreatta et al., 2015) was used for predicting these epitopes and confirmed by IEDB (Sturniolo et al., 1999; Nielsen and Lund, 2009; Wang et al., 2010) and TEPITOPE software (Zhang et al., 2012). Three epitopes per HLA-DRB1 were selected for *in vitro* analysis according to highest predicted binding values (Table 1).

**Table 1:** T-epitopes selected *in silico* and *Pv*RON2 *in vitro* binding.

**Peptide code**	**Sequence**	**CoreF**	HLA-DRB1^*^ allele	**NetMHCIIpan 3.0 (%Rank)**	Binding percentage^*^	IC50 μM^*^	**IC50 ratio**
39147	LKPFYSLETMLMANS	FYSLETMLM	DRB1^*^0401	0.3	92.6	4.6	0.2
			DRB1^*^0701	2.5	83.2	26.0	1.1
			DRB1^*^1101	10.0	79.3	23.0	4.8
			DRB1^*^1302	34.0	63.0	79.0	10.6
39148	NVRKFFLNDVSSIRH	FFLNDVSSI	DRB1^*^0401	1.0	83.0	11.9	0.6
			DRB1^*^0701	5.0	79.9	39.7	1.7
			DRB1^*^1101	19.0	73.8	83.4	17.5
			DRB1^*^1302	1.4	94.2	7.4	1.0
39149	DKSFISEANSFRNEE	FISEANSFR	DRB1^*^0401	3.5	85.8	26.3	1.4
			DRB1^*^0701	17.0	42.8	ND	ND
			DRB1^*^1101	26.0	36.5	ND	ND
			DRB1^*^1302	24.0	33.5	ND	ND
39150	QTAFRKFFKKIISLG	FFKKIISLG	DRB1^*^0401	17.0	83.6	33.7	1.7
		FRKFFKKII	DRB1^*^0701	6.5	67.2	51.8	2.2
			DRB1^*^1101	1.2	87.6	83.4	17.5
			DRB1^*^1302	48.0	7.7	ND	ND
39151	KLKYIFKRRKTMKKK	FKRRKTMKK	DRB1^*^0401	37.0	65.3	40.0	2.1
			DRB1^*^0701	6.0	36.0	ND	ND
		YIFKRRKTM	DRB1^*^1101	0.1	78.5	51.1	10.7
			DRB1^*^1302	27.0	13.1	ND	ND
39152	LFYVNLFIMSSLSRK	LFIMSSLSR	DRB1^*^0401	3.0	65.8	2.8	0.1
		FIMSSLSRK	DRB1^*^0701	2.0	11.0	ND	ND
			DRB1^*^1101	1.4	91.8	1.9	0.4
			DRB1^*^1302	27.0	94.7	8.0	1.1
39153	MKLLQHIPANLLENI	LLQHIPANLL	DRB1^*^0401	0.5	61.2	57.0	2.9
			DRB1^*^0701	0.1	85.4	7.5	0.3
			DRB1^*^1101	6.5	72.7	52.1	10.9
			DRB1^*^1302	0.1	91.9	10.7	1.4
39154	LKFIVRGNNLKFLNN	IVRGNNLKF	DRB1^*^0401	11.0	22.2	ND	ND
			DRB1^*^0701	4.5	43.7	ND	ND
		FIVRGNNLK	DRB1^*^1101	3.0	24.9	ND	ND
		IVRGNNLKF	DRB1^*^1302	0.2	90.1	6.5	0.9
39046	NYEIYIASSSNIYLM	YIASSSNIY	DRB1^*^0401	0.8	91.8	28.4	1.5
			DRB1^*^0701	0.3	91.5	23.3	1.0
			DRB1^*^1101	13.0	82.9	120.0	25.2
		IYIASSSNI	DRB1^*^1302	0.2	92.8	29.6	4.0
39047	RGPVNYHFSNYMNLD	VNYHFSNYM	DRB1^*^0401	16.0	59.8	54.5	2.8
		YHFSNYMNL	DRB1^*^0701	10.0	90.4	6.0	0.3
			DRB1^*^1101	37.0	0.0	ND	ND
		VNYHFSNYM	DRB1^*^1302	13.0	37.8	ND	ND
39048	TPIIVKYDNTHAKNR	IIVKYDNTHA	DRB1^*^0401	12.0	90.7	11.9	0.6
			DRB1^*^0701	41.0	10.5	ND	ND
			DRB1^*^1101	24.0	16.8	ND	ND
			DRB1^*^1302	8.5	3.3	ND	ND

*Peptide code and sequence are shown; core and %rank according to NetMHCIIpan 3.0 predicted HLA-DRB alleles and values. Binding assays and IC50 values were obtained from the methodology for all peptides. IC50 was assessed for peptides having ≥50% binding, IC50 values were expressed as ratios (IC50 peptide/IC50 control peptide), and good binders were considered when their ratio was ≤ 10. Specific peptides were selected as those having the lowest ratio value for each allele and a universal peptide had to have the lowest mean ratio value*.

* Data from this study; ND means that a peptide had less than 50% binding so that its IC50 value was not evaluated. %Rank values were considered as follows: weak binders rank ≤ 10 and strong binders ≤ 2. IC50 values were calculated for each control peptide with each DRB1^*^ allele, the controls HA-DRB1^*^0401 IC50 = 19.44 μM; TT-DRB1^*^0701 IC50 = 23.37 μM; HA-DRB1^*^1101 IC50 = 4.77 μM; TT-DRB1^*^1302 IC50 = 7.46 μM. Peptides having a IC50 ratio ≤ 10 were considered good binders.

Bepipred 1.0 (Larsen et al., 2006) and Antheprot 2000 V6.0 (Deléage et al., 2001) were used for predicting B-cell epitopes. Four epitopes were chosen as they agreed with average high Parker antigenicity, hydrophilicity and solvent accessibility values obtained with Antheprot software, and the high values obtained with the Bepipred tool (0.35 default threshold and 75% specificity); such peptides were further used for analyzing humoral responses *in vitro* (Table 2).

**Table 2:** Humoral response to *Pv*RON2 B-cell epitopes.

**B epitope code**	**Amino acid sequence**	**Average response**
39041	YGRTRNKRYMHRNPGEKYKG	0.159 (*SE* = 0.026)
39042	KLQQEQNELNEEKERQRQEN	0.104 (*SE* = 0.016)
39043	QEQEEEEDDNDPNGSKKNGK	0.142 (*SE* = 0.018)
39044	EKIRKQEEEEEERINNQRRA	0.094 (*SE* = 0.015)
*Pv*GAMA-CT	434–749 aa	0.475 (*SE* = 0.071)

*IgG absorbance readings for individuals exposed to natural P. vivax infection from endemic areas of Colombia. B-cell epitope code, aa sequences, control protein and average response (OD) are shown. SE, standard error*.

### Synthetic peptides

Peptides selected *in silico* were purchased from Twenty First Century Biochemicals Inc. (260 Cedar Hill Street Marlboro, MA 01752 USA) and characterized by matrix-assisted laser desorption/ionization time-of-flight mass spectrometry (MALDI-TOF MS). The biotinylated peptides used as control for HLA peptide binding in competition assays were synthesized using sulfo-NHS-LC-Biotin (Pierce Chemical, Rockford).

### HLA-DR molecules purification

HLA-DRB1^*^ molecules were purified from human HLA-DRB1^*^0401 (IHW09025), HLA-DRB1^*^0701 (IHW09051), HLA-DRB1^*^1101 (IHW09043) and HLA-DRB1^*^1302 (IHW09055) homozygous lymphoblastoid B-cell lines (International Histocompatibility Working Group) and cultured in RPMI-1640 (Gibco) with 10% FBS (Gibco), at 37°C in a 5% CO_2_ atmosphere. The purification was carried out as previously described by Vargas et al. (2003), briefly 5 × 10^9^ cells were lysed at 1 × 10^8^ cell/mL final density in lysis buffer with 10 μg/mL protease inhibitors [antipain, pepstatin A, soybean trypsin, leupeptin, and chymostatin (SIGMA-ALDRICH)]. The lysate was mixed with Protein A-Sepharose CL-4B beads (GE Healthcare) linked to mAb L243 (ATCC HB-55; anti-DR was purified by affinity chromatography using Protein A-Sepharose CL-4B beads) overnight, HLA-DRB1^*^ molecules were obtained by affinity chromatography. HLA-DRB1 protein purity was confirmed by native SDS-PAGE (12%) and Western-blot; positive aliquots' concentration was determined by the Micro BCA protein assay kit (Thermo Scientific); HLA-DRB1 proteins were stored at −80°C until use.

### *In vitro* peptide-binding assays and IC50 values

Peptide binding competition assays were performed to test *Pv*RON2 high-affinity binding peptides selected by *in silico* analysis using NetMHCIIpan 3.0 software. Selected unlabeled peptides competed with biotinylated control peptide in binding to HLA-DRB1^*^. The biotinylated peptides used were haemagglutinin antigen HA_306−318_ (PKYVKQNTLKLAT) for HLA-DRB1^*^04 and HLA-DRB1^*^11 (Hammer et al., 1994; Saravia et al., 2008) and tetanus toxoid (TT) (QYIKANSKFIGITE) for HLA-DRB1^*^07 and HLA-DRB1^*^13 (Doolan et al., 2000).

HLA-DR molecules (0.1 μM) were incubated for 24 h with 5 μM biotinylated HA or TT peptides and a 50-fold excess of unlabeled peptide (250 μM). The mix was incubated for 2 h in Maxisorb NUNC-immune modules (Thermo Scientific) covered with anti-DR. The complex was incubated with alkaline phosphatase streptavidin (Vector Labs) and as substrate alkaline phosphatase yellow (pNPP) liquid substrate (Sigma-Aldrich). Optical density (OD) was determined at 405 nm using a Multiskan GO (Thermo Scientific, Waltham, Massachusetts, USA) ELISA reader. Inhibition was calculated as a percentage, by using the following formula:

$$100\;*\;\left[1-\left(\frac{\Delta OD\;in\;the\;presence\;of\;competitor}{\Delta OD\;in\;the\;absence\;of\;competitor}\right)\right]$$

IC50 values (50% concentration inhibition) were determined for peptides able to inhibit high-affinity control peptide binding to a particular HLA-DR by more than 50% (Saravia et al., 2008). The peptides and control peptides were tested in 5–250 μM serial dilutions for the competition assays; Mathematica (version 10.1) software (Wolfram Research, Inc., Mathematica, Champaign, IL 2015) was used for calculating IC50 values, using two-phase exponential decay. IC50 values were calculated as a relative value using the following formula:

$$1-\left[\frac{\left(\Delta OD\;in\;the\;presence\;of\;competitor\right)}{\left(\Delta OD\;in\;the\;absence\;of\;competitor\right)}\right]$$

### Study population

Peripheral blood was obtained from 79 people living in the Colombian departments of Chocó and Córdoba (known *P. vivax* malaria endemic areas, having the highest case incidence) who had suffered previous episodes of malaria. Inclusion criteria consisted of being over 18 years-old, residing in a *P. vivax*-endemic area, having had 1 or more episodes of *P. vivax* malaria (the last one 6 months beforehand) and having received suitable treatment for the disease. Although a stronger immune response would have been expected in acutely-infected *P. vivax* individuals, the construction of study groups required a prior HLA typing and thus, a second sample had to be taken from individuals matching the alleles of interest to assess antigenicity. Taking this into account, the antigenicity sample was taken from people that had suffered *P. vivax* malaria at least 6 months earlier. A control group of 50 individuals was selected; this consisted of healthy adults residing in Bogotá, Colombia, who had never lived in malaria-endemic areas and who had never experienced malarial infection. This study was performed according to the legal framework for research in Colombia and Ministry of Health's Resolution 8430 of 1993. The patients had the least risk, all data were kept confidential and were rigorously protected. The samples were collected after all individuals signed an informed consent form; all procedures were evaluated and approved by FIDIC's ethics committee.

### HLA-DRB1 typing

Genomic DNA (gDNA) from 300 μL peripheral blood samples was extracted using a Wizard Genomic DNA Purification Kit (Promega Corporation, Madison, USA), following the manufacturer's instructions. gDNA was used for high resolution HLA-DRB1 typing by Histogenetics (Ossining, NY, USA) through Next Generation Sequencing (NGS) technology using Illumina MiSeq.

### PBMC isolation

Twenty-nine people carrying HLA-DRB1 typing for HLA-DRB1^*^04, HLA-DRB1^*^07, HLA-DRB1^*^11 and HLA-DRB1^*^13 alleles were selected from *P. vivax* endemic areas of Colombia's Córdoba and Chocó departments. Eight people carrying the same HLA-DRB1^*^ alleles from a non-endemic area formed the control group. About 40 mL peripheral blood was collected in citrate phosphate dextrose (CPD) tubes and 6 mL peripheral blood in BD vacutainer serum collection tubes (BD Vacutainer Oakville, ON). Thick blood smears were used for confirming samples negative for malaria. Peripheral blood mononuclear cells (PBMC) were isolated by Ficoll-Paque PLUS (GE Healthcare) gradient centrifugation. Briefly, the buffy coat was resuspended in RPMI 1640 (Gibco) and separated by Ficoll, spinning at 1,000 g for 30 min at room temperature (RT). Mononuclear cells were collected, washed and spun at 800 g for 10 min, twice. Cell viability was evaluated by trypan blue exclusion test and cells were counted in a Neubauer chamber.

### T-cell proliferation

Briefly, 2 × 10^5^ PBMC were cultured in 200 μL RPMI-1640 (Gibco), 2 mM glutamine, 1 mM sodium pyruvate, 2 g/L sodium bicarbonate, 100 μg/mL streptomycin and 100 U/mL penicillin (all Gibco) and 10% heat-inactivated autologous plasma in 96-well round-bottomed plates (Costar, Corning Incorporated). Proliferation activity was evaluated by flow cytometry using carboxyfluorescein diacetate *N*-succinimidyl ester (CFSE, 5μM) (CellTrace CFSE cell proliferation kit, Molecular Probes, Eugene, Oregon, USA) reduction in replicating cells.

The cells were left without stimulation (unstimulated control) or were stimulated by co-culture with synthetic peptides (10 μg/mL) or 2% mitogen phytohemagglutinin (PHA) (Sigma) or 5 μg/mL *P. vivax* lysate as positive controls. *Pv*12 low binding peptide was selected (39115) by binding assay and used as negative control (manuscript in preparation). The 96-well plates were incubated in 5% CO_2_ at 37°C for 5 days; 100 μL culture supernatant was then collected per well and stored at −80°C until analysis for cytokine production. Duplicate assays were carried out.

The CD4-Pacific Blue-stained cell stimulation index was calculated by proliferative cells' relative percentage loss of carboxyfluorescein succinimidyl ester (CFSE) in the presence of antigen, divided by percentage relative CFSE loss for proliferative cells without antigen (Racanelli et al., 2011). Data was averaged for each antigen and for both exposed individuals and control groups. SI ≥ 2 was taken as antigen-specific positive proliferation. A Pacific Blue-labeled mouse anti-human CD4 (RPA-T4 clone) antibody (BD Biosciences), was used for CFSE-cell cluster measure. The samples were then read on a FACS Canto II flow cytometer; FlowJo software (v7.6.5, Ashland, Oregon, USA) was used for analyzing the results.

### Cytokine secretion

IFN-γ, TNF, IL-10, and IL-6 levels in lymphocyte culture supernatant were determined with a BD CBA Human Th1/Th2 Cytokine Kit II (San Jose, CA, USA), following the manufacturer's instructions. Supernatants were read on a FACS Canto II flow cytometer; FCAP Array software (v3.0.1) was used for analyzing the results. Results were expressed in pg/mL for each cytokine; data were compared between unstimulated and stimulated PBMC supernatant culture. Two standard deviations higher than that for control group were taken as positive antigen-specific production.

### Indirect immunofluorescence assays (IFA)

IFA followed that previously described by Moreno-Pérez et al. (2013) with some modifications. Briefly, blood samples from individuals having active *P. vivax* infection were spun at 1,750 g for 12 min at RT. Both plasma and buffy coat were recovered and parasite red blood cells (pRBC) were washed with saline solution. pRBC were passed through a 60% Percoll gradient and spun at 1,750 g for 20 min. *P. vivax*-pRBC were diluted until 5–7 schizonts per field and confirmed by acridine orange. Twenty μL of diluted pRBC were placed into multitest microscope slide wells (Tekdon Incorporated) and incubated for 30 min. The supernatant was then removed, and microscopic slides left overnight (ON) at room temperature (RT) to air dry. The slides were then blocked for 30 min at RT with tris-buffered saline (TBS) 1% bovine serum albumin (TBSA) solution and washed three times. The serum samples from 30 exposed individuals and 8 sera from the control group were diluted in TBSA at 1:50 dilution and incubated for 1 h in a humid chamber. Reactivity was observed by fluorescence microscopy using anti-human IgG-FITC antibody (Sigma-Aldrich) diluted 1:50 in TBSA for 45 min in a humid chamber. The parasite nuclei were stained with 4′, 6-diamidino-2-phenylinodole dihydrochloride (DAPI) (0.25 μg/mL) for 5 min at RT and washed twice with 0.05% TBS-Tween 20 and three washes with TBS to remove excess reagent. The slides were visualized on an Olympus BX51 fluorescence microscope, using 100X oil immersion objective; DP2-BSW software (v2.2 Olympus Corporation) was then used to take images and ImageJ 1.51n software (National Institutes of Health, USA) for merging images.

### Enzyme-linked immunosorbent (ELISA) and subclass IgG assays

Total IgG antibodies were measured in serum from exposed individuals and control group. Maxisorb NUNC-immune modules (Thermo Scientific) were coated with 1 μg of each epitope and of r*Pv*GAMA (10 μg/mL) (Baquero et al., 2017) in phosphate-buffered saline (PBS), pH 7.2, and incubated ON at 4°C. The immune modules were washed three times with PBS-0.05% Tween 20 solution (PBST) the next day and then blocked with 2.5% (wt/vol) non-fat powdered milk in PBST solution for 1 h at RT. Serum at 1:100 dilution was incubated for 2 h (100 μL per well) in duplicate. Secondary antibody horseradish peroxidase-conjugated goat anti-human IgG (Vector labs) was added at 1:10,000 dilution in blocked solution and incubated for 1 h at RT. TMB 2-Component Microwell Peroxidase Substrate (Sera-Care) was added at 100 μL/well to detect monoclonal antibody binding. The reaction was stopped by adding an equal volume of 1M phosphoric acid (H_3_PO_4_); OD was measured at 450 nm using a Multiskan GO (Thermo Scientific, Waltham, Massachusetts, USA) ELISA reader. Cut-off value was determined as negative control serum samples' mean plus two standard deviations; IgG subclasses were determined for positive total IgG serum.

The ELISA protocol described above was followed to evaluate IgG subclasses with minor modifications, as follows: 3% (wt/vol) bovine serum albumin (BSA, Sigma) in PBST was used as blocking agent/solution and serum at 1:100 dilution was incubated for 2 h in duplicate. A 1:1,000 dilution of monoclonal anti-human IgG1–biotin antibody produced in mouse (clone 8c/6-39), 1:15,000 monoclonal anti-human IgG2–biotin antibody produced in mouse (clone HP-6014), 1:40,000 monoclonal anti-human IgG3–biotin antibody produced in mouse (clone HP-6050) and 1:60,000 anti-human IgG4–biotin antibody, mouse monoclonal (clone HP-6025) (Sigma Aldrich) were used. ImmunoPure streptavidin, horseradish peroxidase conjugate (Thermo Scientific), at 1:5,000, dilution, was used as secondary antibody and TMB 2-Component Microwell peroxidase as substrate. The reaction was stopped by adding an equal volume of 1M phosphoric acid (H_3_PO_4_). OD was measured at 450 nm, using a Multiskan GO ELISA reader (Thermo Scientific, Waltham, Massachusetts, USA).

### Statistical analysis

GraphPad Prism software (version 5.0, San Diego, CA, USA) was used for analysis and constructing graphs. A Mann-Whitney test was used for comparing two groups regarding non-parametric data and Kruskal-Wallis test (with Dunn's multiple comparison post-test) for comparing more than two groups. Student's *t*-test was used for comparing two groups of data having a normal distribution. A 95% confidence interval was used. *p* ≤ 0.05 was considered significant. Significance level has been highlighted on all graphs by asterisks, as follows: ^*^p < 0.05; ^**^p < 0.005, and ^***^p < 0.0005.

## Results

### T-epitope selection according *in vitro* binding profile

**Figure 1:**
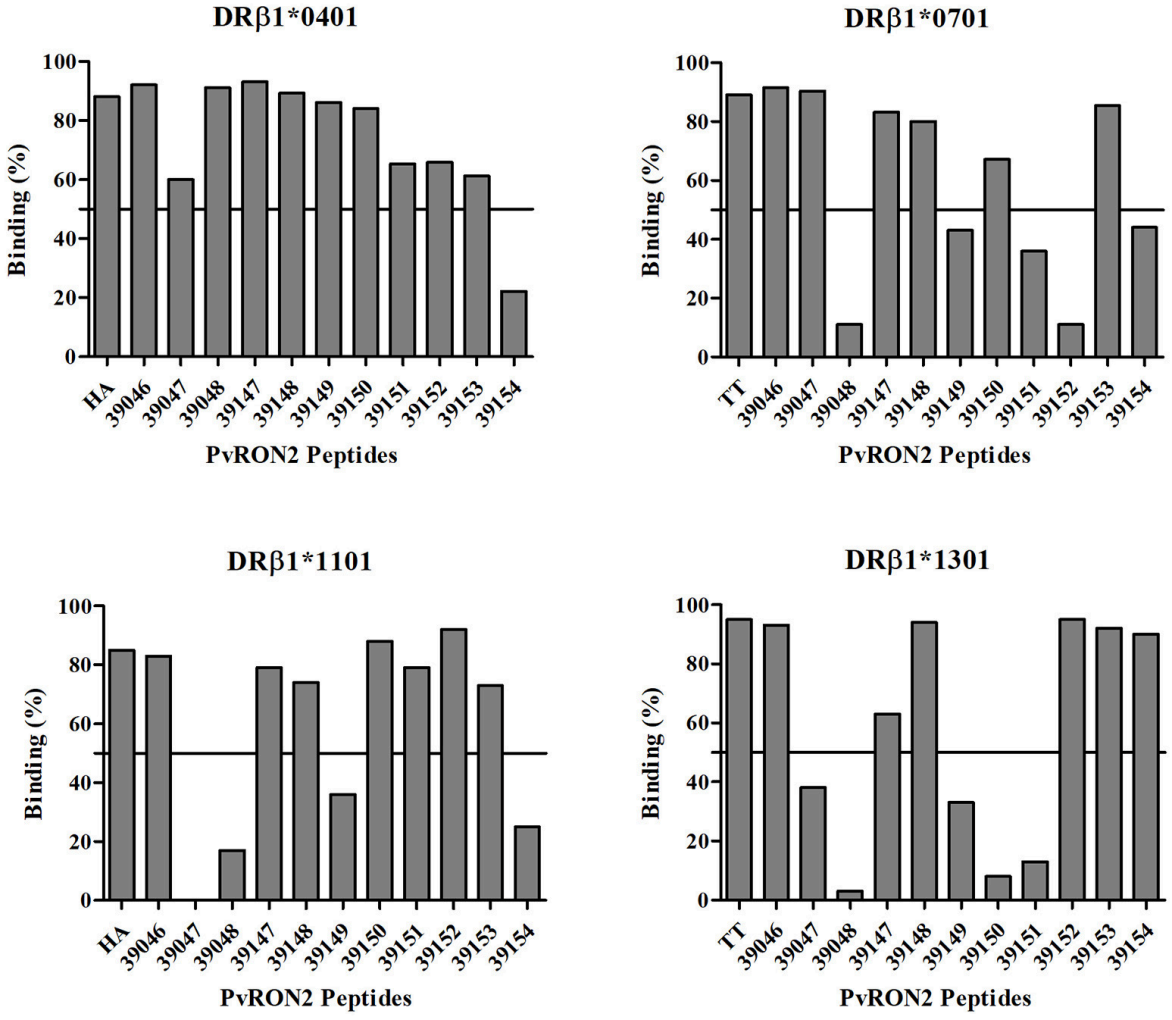
*Pv*RON2 peptides *in vitro* binding to purified HLA-DRB1* molecules. A cut-off line is shown at 50% binding, used for selecting high-binding peptides for further evaluation of IC50 value. Each plot shows percentage epitope binding to HLA-DRB1* in this study and that for their control peptide.

Table 1 gives *Pv*RON2 antigenic epitope prediction results. Eleven epitopes were selected *in silico* for HLA-DRB1^*^04, ^*^07, ^*^11, and ^*^13 alleles (3 epitopes for each allele and 2 epitopes for the ^*^07 allele). These epitopes were evaluated *in vitro* for their ability to bind all alleles of interest; those having greater than 50% binding were carefully chosen as high-binding peptides (Figure 1).

*In vitro* results showed that 10/11 (90.9%) peptides bound to the HLA-DRB1^*^04 allele (73.45% mean binding), being the most promiscuous allele studied; 7/10 (70%) peptides bound to HLA-DRB1^*^11 (64.47% mean binding); 6/11 (54.5%) of the peptides bound to HLA-DRB1^*^07 (58.33% mean binding) and HLA-DRB1^*^13 (56.55% mean binding). Four of the eleven peptides bound to all alleles studied here and were thus considered universal epitopes (39046, 39147, 39148, and 39153). Experimental binding assays and *in silico* binding predictions agreed in a 70.45% for the alleles studied (Table 1).

**Figure 2:**
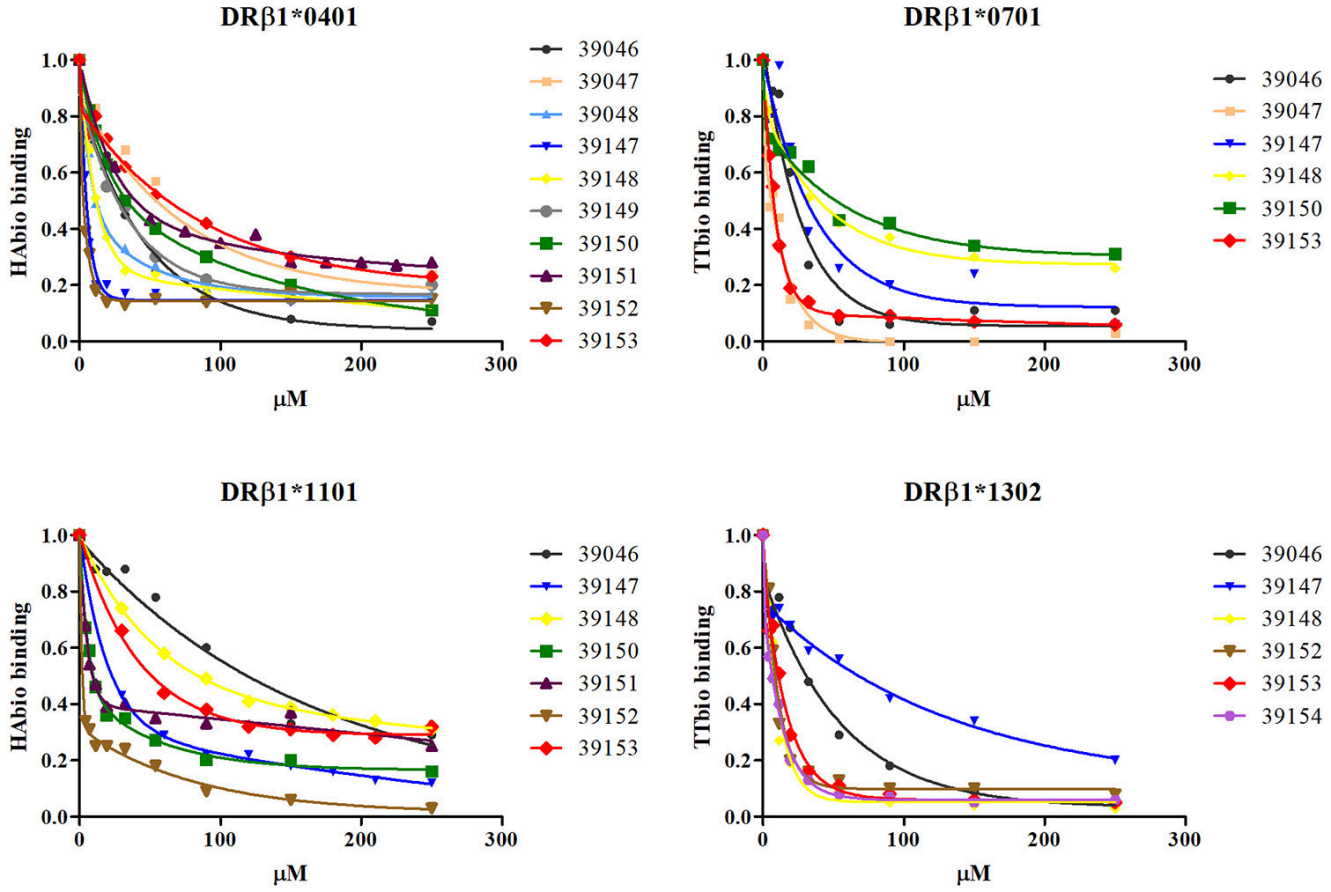
*In vitro* assays for calculating a *Pv*RON2 peptide's IC50 value. Different epitope concentrations were evaluated for calculating the value at which control peptide was displaced by 50% (using a second order exponential decay function). Each point under the curve represents evaluated epitope concentration-dependent control peptide (μM) binding.

The IC50 value was calculated for all high-binding peptides to select the ones displaying higher affinity. Different peptide concentrations (μM) were used and the point at which 50% of the control peptide was displaced was thus calculated. IC50 μM value was calculated using a second order exponential decay function (Figure 2). IC50 assays demonstrated that epitope 39152 had the lowest IC50 ratio for HLA-DRB1^*^04 (0.15) and HLA-DRB1^*^11 (0.4), so it was thus selected as a good epitope for both alleles. Epitope 39047 (0.26) was selected as specific epitope for HLA-DRB1^*^07 and epitope 39154 (0.88) for HLA-DRB1^*^13. Of the four universal epitopes, peptide 39153 had the lowest IC50 mean value (Table 1, Figure 2). The selected epitopes were screened for antigenicity according to their HLA-DRB1^*^ binding profile in previously typed patients.

### Evaluating T-cell response against selected epitopes

The people in the study had to have resided in the area for at least the last 5 years (average 29 years) and have had 1 or multiple episodes of *P. vivax* malaria, the last episodes dated between 2011 and 2015. It is well known that a naturally-acquired response requires a long period of time and multiple exposures to the parasite (Wipasa et al., 2002). HLA-DRB1^*^ allele distribution for the 79 people here typed is shown in the Supplementary Table 1. Among the alleles of interest, HLA-DRB1^*^04 had a frequency of 12.66%, HLA-DRB1^*^07 a frequency of 12.03%, HLA-DRB1^*^11 a frequency of 8.23% and HLA-DRB1^*^13 a frequency of 9.49%.

PBMC from individuals exposed to *P. vivax* infection (and control group) were stimulated with 10 μg/mL *Pv*RON2 peptides and incubated for 5 days to evaluate proliferative response. Both universal epitope 39153 and DRB1^*^ allele-specific peptides induced proliferation (≥2 Stimulation Index) in individuals from endemic areas, whereas there was no proliferation regarding specific peptides for each allele or parasite lysate in the control group (Table 3 and Supplementary Table 2). There were statistically significant differences for universal epitope 39153 (*p* = 0.0075), DRB1^*^13-specific peptide 39154 (*p* = 0.0387) and DRB1^*^07-specific peptide 39047 (*p* = 0.0260) between the group of exposed individuals and the control group. This response to the different antigens used in the lymphoproliferation assay, was compared with a Kruskal-Wallis test (with a Dunn's multiple comparison post-test), where no statistically significant differences were found in response to the different peptides.

**Table 3:** A summary of PBMC proliferative response to *Pv*RON2 T-cell epitopes.

**Antigen**	HLA-DRB1^*^	**Average response (SI)**		***p*-value**
		**Exposed individuals**	**Control group**	
39153	Universal epitope	3.354 (SE 0.578)	0.935 (SE 0.282)	0.0075^**^
39152	DRB1^*^04 DRB1^*^11	3.052 (SE 1.09)	1.004 (SE 0.198)	0.1751
39047	DRB1^*^07	3.108 (SE 0.737)	1.156 (SE 0.267)	0.0260^*^
39154	DRB1^*^13	3.697 (SE 1.17)	0.888 (SE 0.327)	0.0387^*^
39115	Low-binding control peptide	2.018 (SE 0.347)	0.87 (SE 0.231)	0.0289^*^
Parasite lysate	Positive control	3.664 (SE 0.607)	1.474 (SE 0.255)	0.0735

*Data regarding individuals exposed to P. vivax infection and control group. (SI, stimulation index; SE, standard error). Mann-Whitney's test and Student's t-test p-values were determined for exposed individuals compared to control group*.

* *p < 0.05*,

** *p < 0.005*.

**Figure 3:**
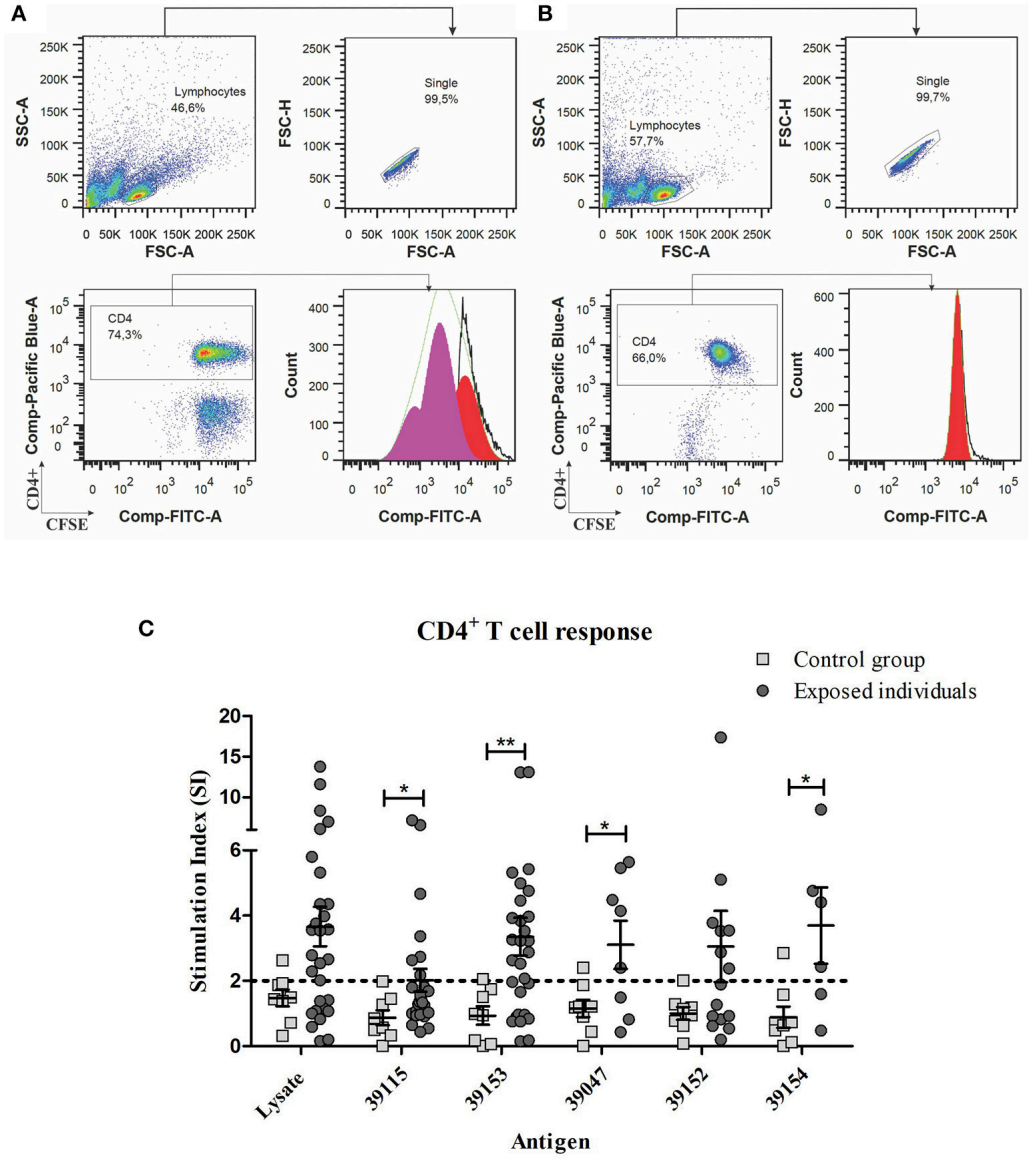
Gating strategy for the proliferation assays and PBMC proliferative response to *Pv*RON2 epitopes from individuals exposed to *P. vivax* infection compared to control group. **(A)** *P. vivax*-exposed individuals' PBMC stimulated with universal peptide (39153). **(B)** Non-stimulated *P. vivax* exposed individuals' PBMCs. **(A,B)** Upper left plot, selected lymphocyte population (SSC-A vs. FSC-A), upper right plot selection of single cells from lymphocyte population (FSC-H vs. FSC-A). The lower left-hand plot shows gated CFSE label lymphocytes (Comp-FITC-A) for analyzing CD4+ T-cells (Comp-Pacific Blue-A). Lower right-hand plot shows CD4+ lymphocyte proliferation analyzed by FlowJo software (v7.6.5, Ashland, Oregon, USA) using 7 peaks or cell generations. **(C)** Mann-Whitney and Student's *t*-tests were used for assessing statistically significant differences between exposed individuals and control group. Universal peptide 39153 (*n* = 29), DRB1*04 and DRB1*11 peptide 39152 (*n* = 15), DRB1*07-specific peptide 39047 (*n* = 8), DRB1*13-specific peptide 39154 (*n* = 6), low-binding control peptide 39115 (*n* = 29) and *P. vivax* lysate (*n* = 29) responses are shown. The CD4+ cells were labeled with Pacific Blue mouse anti-human CD4 (RPA-T4 clone) antibody. Statistically significant differences (*p* ≤ 0.05) are shown and data represents the means ± SEM for all values. *p < 0.05 and **p < 0.005.

Low-binding control peptide 39115 showed the lowest proliferative response (SI = 2.018) and was significantly different regarding the control group (*p* = 0.0289). *P. vivax* lysate also induced a greater proliferative response in exposed individuals, despite no statistically significant differences were observed when compared to the control group (Figure 3). However, peptide 39115 induced T cell proliferation in 7 of the 29 exposed individuals, despite no binding to HLA-DRB1^*^ was either predicted or observed *in vitro*. Considering that this peptide is a T epitope from the *Pv*12 protein as shown by the lymphoproliferation assays, further analyses to assess whether it is being presented by other class II molecules such as HLA-DP or HLA-DQ are worth carrying out.

*P. vivax* lysate also induced a greater proliferative response in exposed individuals; however, no statistically significant differences were observed when compared to control group (Figure 3). The mean SI = 12.47 ± 1.636 SE is for exposed individuals and mean SI = 5.45 ± 1.138 SE for the control group. Despite the SI values of PMBCs stimulated with PHA were significantly higher between exposed individuals and control group (*p* = 0.0103), 96.6% of exposed individuals and the 100% of control group responded to PHA (data not shown).

### *Pv*RON2 epitope-dependent cytokine secretion

**Figure 4:**
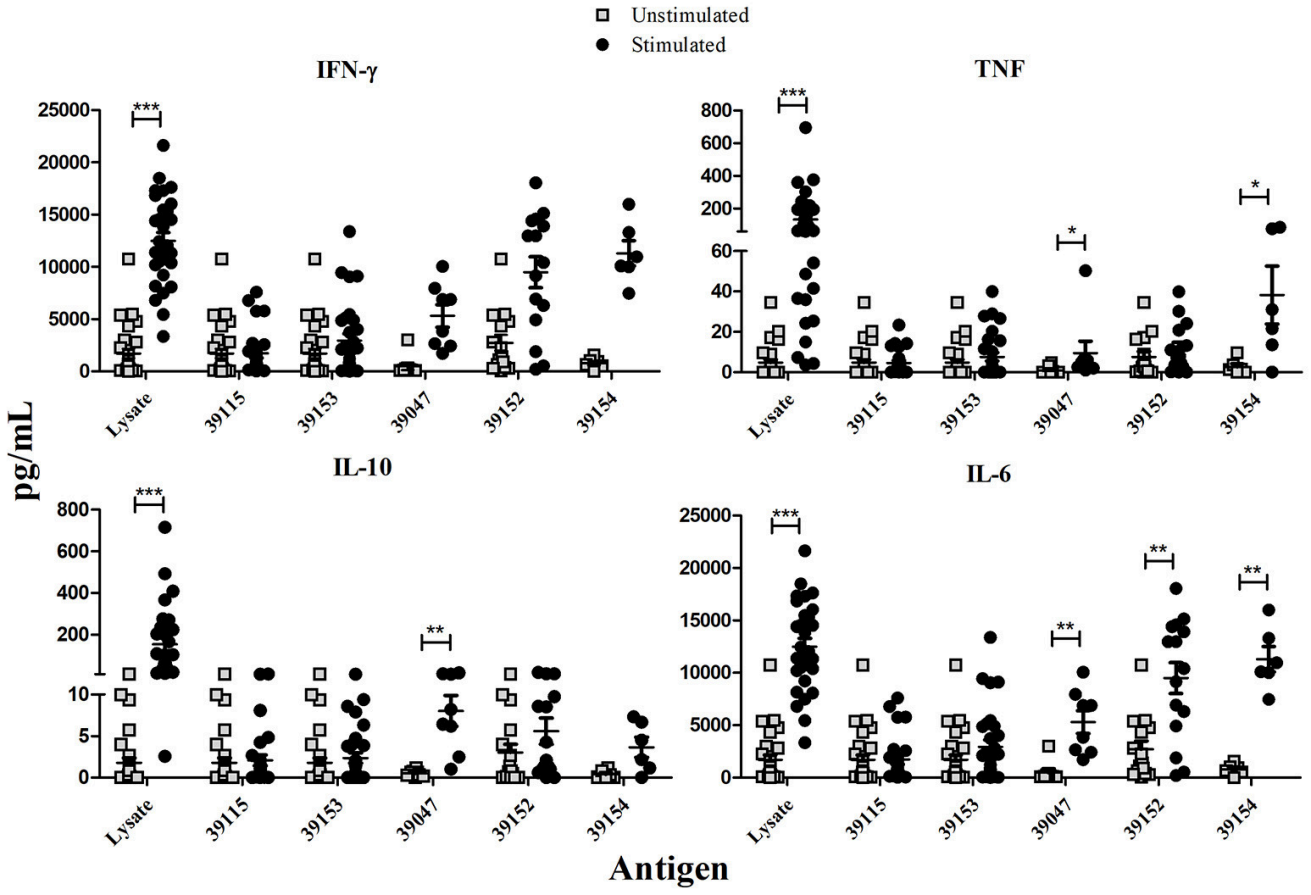
Exposed individuals' supernatant culture *in vitro* cytokine production. Individual data shows the mean value of non-stimulated and PBMCs stimulated with universal epitope (39153), specific epitopes 39047, 39152, and 39154, and *P. vivax* lysate. IFN-γ, TNF, IL-10, and IL-6 levels were measured by CBA kit; cytokine concentration is expressed in pg/mL. Statistically significant differences (*p* ≤ 0.05) are shown and data represents the means ± SEM for all values. *p < 0.05, **p < 0.005 and ***p < 0.0005.

IFN-γ, TNF (Th1 profile) and IL-10, IL-6 (Th2 profile) production in culture supernatant was quantitatively measured after stimulating PBMCs with peptides selected for HLA-DRB1^*^ by binding assays. *P. vivax*-lysate and PHA were used as positive controls and unstimulated PBMCs as a baseline. Statistical analysis between unstimulated and stimulated PBMCs from exposed individuals showed that IFN-γ was only significant after stimulation with *P. vivax*-lysate (*p* = 0.0001). TNF production was significantly different for peptides 39047 (*p* = 0.01), 39154 (*p* = 0.04) and *P. vivax*-lysate (*p* = 0.0001). IL-10 had higher production with peptide 39047 (*p* = 0.001) and *P. vivax*-lysate (*p* = 0.0001). IL-6 responses were significantly greater to peptides 39047 (*p* = 0.01), 39152 (*p* = 0.0025), 39154 (*p* = 0.002) and *P. vivax*-lysate (*p* = 0.0001) (Figure 4). Cytokine levels were compared with the Kruskal-Wallis test (with a Dunn's multiple comparison post-test) between 39115 and all other peptides in exposed individuals, a significantly higher TNF and IL-6 production was observed for peptide 39154 (*p* < 0.0001).

Control group data was also analyzed; significant differences were found for IFN-γ production after PBMCs had been stimulated with peptides 39047 (*p* = 0.002), 39154 (*p* = 0.0006) and *P. vivax*-lysate (*p* = 0.0006). IL-6 production was greater after being stimulated with peptides 39152 (*p* = 0.0006), 39047 (*p* = 0.002), 39154 (*p* = 0.01) and *P. vivax*-lysate (*p* = 0.0001). This suggests that naïve T-cells and/or other innate cells, such as macrophages, natural killer (NK), natural killer T-cells (NKT) and non-cytotoxic innate lymphoid cells recognized peptides and lysate and produced cytokines (Artis and Spits, 2015) (Supplementary Figure 1).

**Figure 5:**
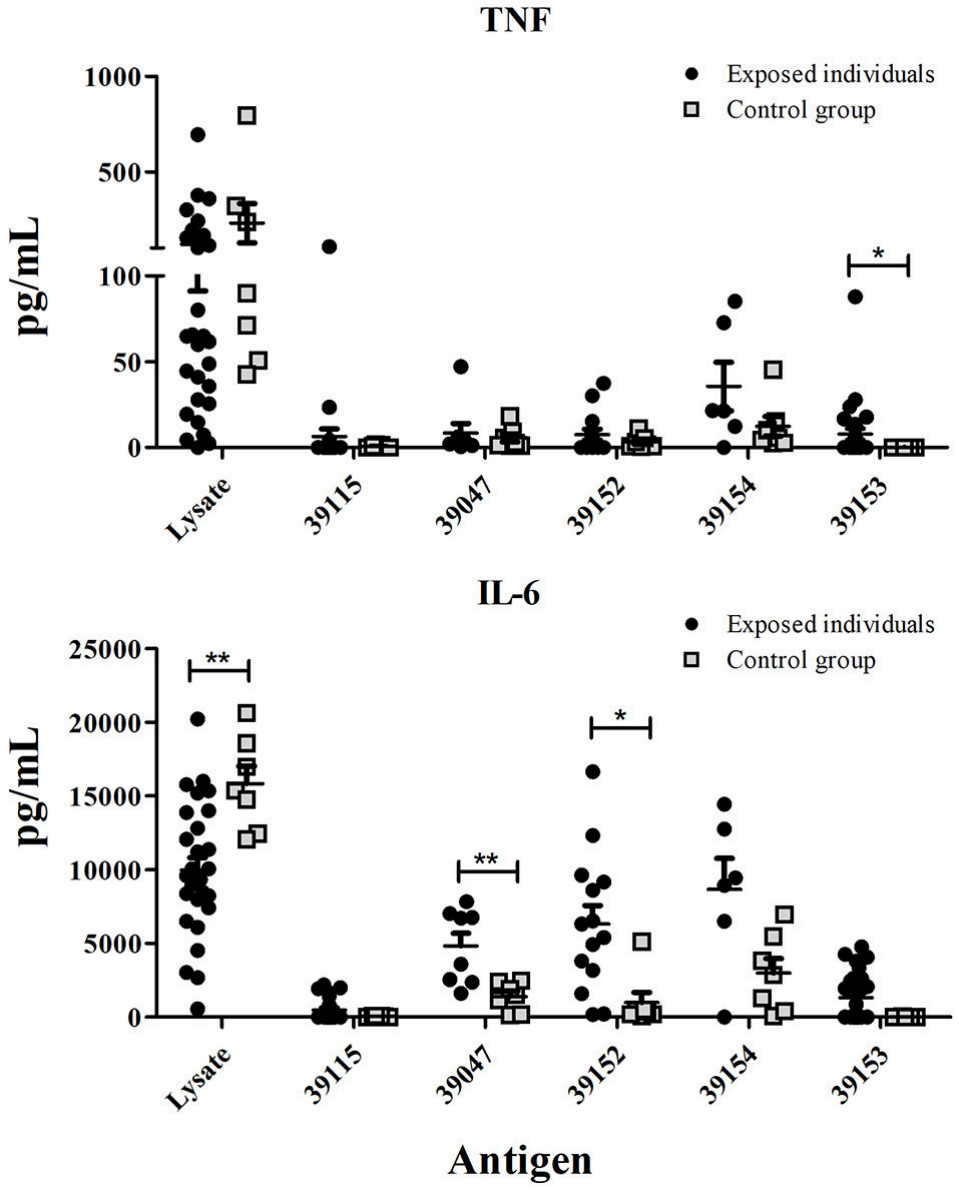
Exposed individuals and control group supernatant culture *in vitro* cytokine production. Individual cytokine values from PBMCs stimulated with universal peptide (39153), specific epitopes 39047, 39152, and 39154, and *P. vivax* lysate. TNF and IL-6 levels were measured by CBA kit and cytokine concentration is expressed in pg/mL. Statistically significant differences (*p* ≤ 0.05) are shown and data is the means ± SEM for all values. *p < 0.05 and **p < 0.005.

Cytokine levels were compared between exposed individuals and control group, significant differences being found regarding TNF production for epitope 39153 (*p* = 0.0127). Significant differences were found for epitope 39047 (*p* = 0.005) and 39152 epitope (*p* = 0.010) IL-6 production (Figure 5).

### Detecting antibodies against *P. vivax*-infected RBC by IFA

Naturally-acquired anti-malarial antibodies were detected using Multitest slides (MP Biomedicals) coated with parasitized RBC. The thirty exposed patients' sera reacted against *P. vivax* but the control group's sera did not. Supplementary Figure 2 shows the fluorescence pattern obtained with these sera.

### An analysis of *Pv*RON2 B-epitope humoral immune response in *P. vivax*-exposed individuals

**Figure 6:**
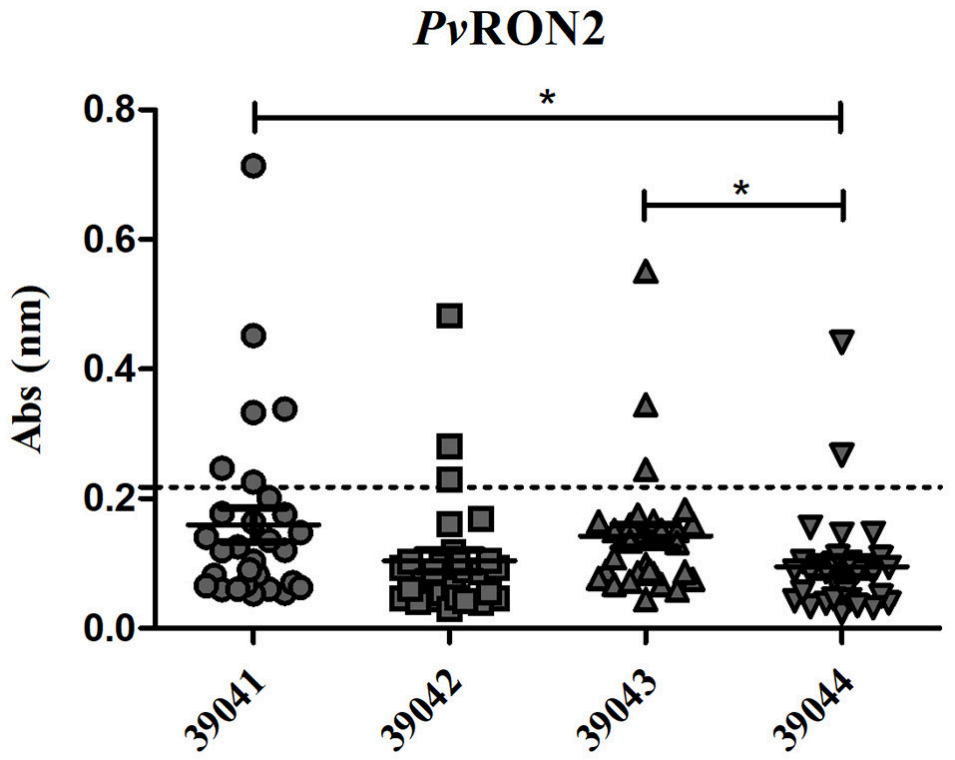
IgG antibody response against four *Pv*RON2 B-cell epitopes (*n* = 30). Seropositive samples were those above the cut-off point (0.218, dotted line), calculated as control group's mean plus two standard deviations. The Kruskal-Wallis test was used for analyzing differences between each B-epitopes response in *P. vivax*-exposed individuals' samples. *p < 0.05.

Antibody response against four *in silico* selected *Pv*RON2 B-epitopes was evaluated in sera from 30 individuals exposed to natural *P. vivax* infection (Figure 6, Table 2). The highest number of seropositive samples was for the 39041-epitope (6/30, 20% of samples), followed by 39042 and 39043 (3/30, 10% of samples) and 39044 (2/30, 6.6% of samples). Peptides 39041 and 39043 showed the highest mean response (0.1598 and 0.1422, respectively), and significant differences were observed regarding 39044 which had the lowest mean response (0.0949) out of all four peptides. The Kruskal-Wallis test (with Dunn's multiple comparison post-test) was used for statistical analysis, there were significant differences regarding epitope response (*p* = 0.003). Although there were no statistically significant differences between exposed individuals and control group, there was a tendency for a greater response in the first group (data not shown). r*Pv*GAMA was used as positive control, 70% of samples being seropositive (21/30 samples); significant differences with control group were observed (*p* = 0.0004) (Supplementary Figure 3).

**Figure 7:**
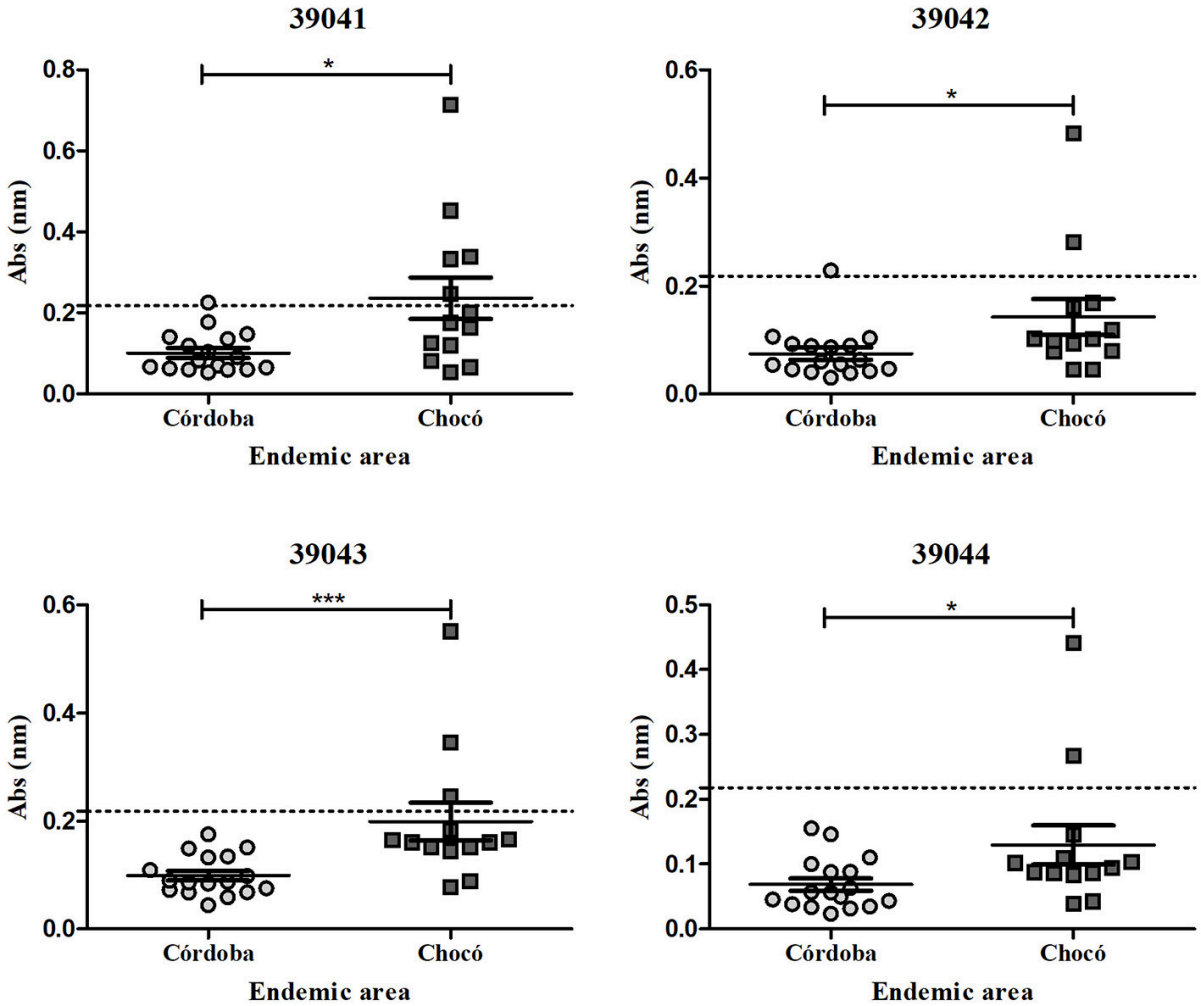
IgG antibody response to *Pv*RON2 B epitopes by endemic area. Significant differences (calculated by Mann-Whitney test) between samples from Colombia's Chocó (*n* = 13) and Córdoba (*n* = 17) departments are shown. The dashed line indicates the cut-off point for seropositive samples. *p < 0.05 and ***p < 0.0005.

The ELISA results were analyzed by endemic area, showing an evident tendency for a greater response in the samples from the Chocó department compared to the Córdoba department (Figure 7). The Mann-Whitney test gave a significantly higher response for peptides 39041 (*p* = 0.0135), 39042 (*p* = 0.0171), 39043 (*p* = 0.0007) and 39044 (*p* = 0.0492) in *P. vivax*-exposed individuals. Samples from Colombia's Chocó (*n* = 13) and Córdoba (*n* = 17) departments. From these samples, six reacted positively to at least one *Pv*RON2 B-epitope, where 83% (*n* = 5) of the samples were from Chocó's department. Positive sera reacted to the four peptides, 83% were from samples from the Chocó department. Although 39041, 39042, and 39044 peptides had no significant differences between exposed individuals from the Chocó and control group, 39043 had a significantly higher response (*p* = 0.0186). Differences between endemic areas were only observed in response to *Pv*RON2 B-cell epitopes since there were no significant differences between both areas regarding *Pv*GAMA (*p* = 0.4265), as it was expected to occur when a whole recombinant protein is used as antigen (several epitopes present within it, could be differentially recognized by exposed individuals, and a similar overall response was thus detected). Seropositive samples were selected for evaluating IgG subclasses.

### The prevalence of IgG subclass response against *Pv*RON2 B-epitopes in individuals exposed to natural *P. vivax* infection

**Figure 8:**
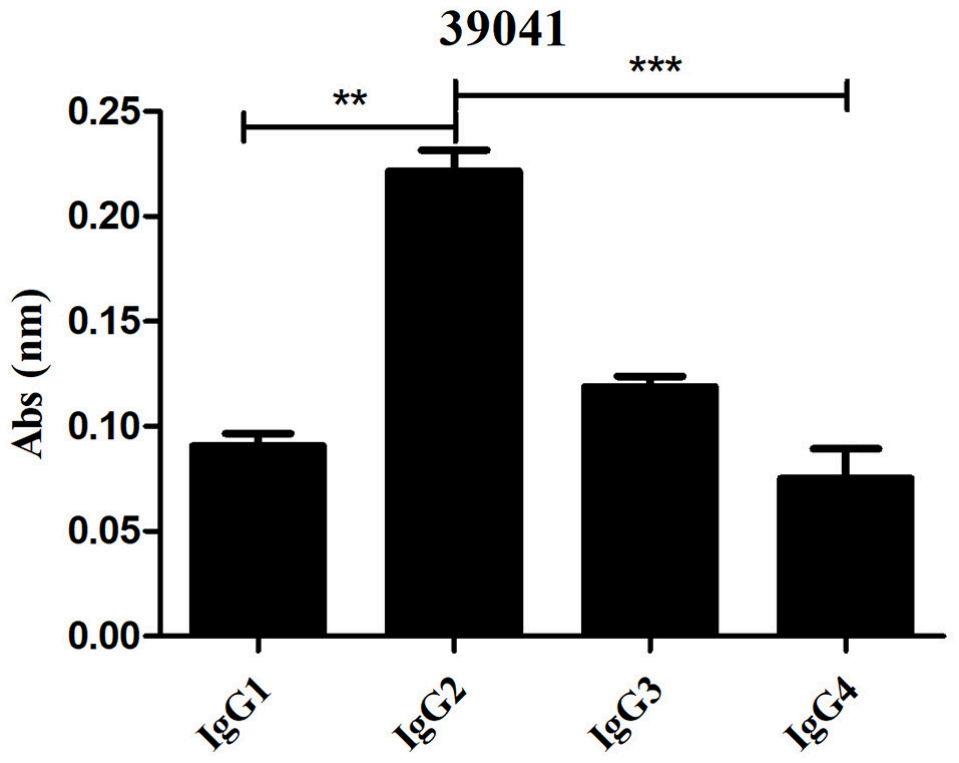
Evaluating IgG subclass response to the 39041 epitope (*n* = 6). The Kruskal-Wallis test was used for analyzing differences between each IgG subclass response in *P. vivax*-exposed individuals' samples. **p < 0.005 and ***p < 0.0005.

Seropositive samples from exposed individuals recognizing B-epitopes were selected for IgG subclass evaluation. Of the four peptides evaluated, only 39041 had significant differences between IgG subclasses (*p* = 0.0004) while there were no significant differences for the other peptides. 39041 had clear IgG2 predominance regarding other subclasses, having statistically significant differences with IgG1 and IgG4 (the latter having the lowest mean response) (Figure 8).

## Discussion

Developing countries desperately need strategies aimed at preventing malaria (especially that caused by *P. vivax*), such as approaches for developing specific drugs and protective vaccines which are currently unavailable. Although there are *P. vivax* vaccines in phases I and IIa (López et al., 2017), they have not induced sterile protection (Bennett et al., 2016). Developing a *P. vivax* vaccine requires studies analyzing naturally-infected patients' immune response regarding proteins involved in erythrocyte invasion. Several *P. vivax* vaccine candidates' binding regions have been characterized to date, such as reticulocyte binding proteins (RBPs) (Urquiza et al., 2002), the Duffy binding protein (DBP) (Ocampo et al., 2002), the *P. vivax* GPI-anchored micronemal antigen (*Pv*GAMA) (Baquero et al., 2017), some proteins from the tryptophan-rich antigen (*Pv*TRAg) family (Zeeshan et al., 2014), merozoite surface protein-1 (*Pv*MSP-1) (RodríGuez et al., 2002) and apical membrane antigen-1 (AMA-1) (Arévalo-Pinzón et al., 2017). Important mediators in AMA-1 erythrocyte binding have also been identified, such as rhoptry neck proteins (RONs), including *Pv*RON5 (Arévalo-Pinzón et al., 2015), *Pv*RON4 (Arévalo-Pinzón et al., 2013) and *Pv*RON2 (Arévalo-Pinzón et al., 2011). RONs have been strongly associated with MJ formation, thereby helping parasite entry into erythrocytes; RON2 is a vaccine candidate since anti-AMA1 and anti-RON2 antibodies can block erythrocyte invasion (Arévalo-Pinzón et al., 2011; Lamarque et al., 2011; Srinivasan et al., 2011; Tyler et al., 2011; Vulliez-Le Normand et al., 2017).

The data presented here has described naturally-acquired T-cell immune responses to *Pv*RON2 high-affinity binding-MHC-II DRB1^*^ peptides in *P. vivax*-exposed individuals from two endemic areas of Colombia. Certain requirements are involved in protection-inducing vaccine design; for example, the proper antigen presentation by MHC-II and MHC-I molecules to T-cell receptors, so as to induce strong immune responses. MHC class II and I molecules are critical in host–pathogen interactions as they determine host immune response quality. High-affinity peptide-MHCII-TCR binding time or peptide-MHCII complex amount is critical for mounting protective T-cell responses (Blum et al., 2013; Tubo et al., 2013). Post-genomic era bioinformatics tools and reverse vaccinology approaches have drawn scientists' attention, since an individual antigen can be screened from one or several microorganisms and the highest affinity epitopes be determined from these might induce protective immune responses (Sette and Rappuoli, 2010).

This study has analyzed the *Pv*RON2 sequence aa to determine high-binding HLA-DRB1^*^ T-cell epitopes *in silico*. NetMHCIIpan 3.0 (Andreatta et al., 2015) predicted eleven high-affinity HLA-DRB1^*^ epitopes where at least one epitope bound to one HLADRB1^*^ molecule; this was confirmed by *in vitro* competition assays using biotin-control peptides. However, some predicted epitopes have not bound to HLA-DRB1^*^, according to other studies (Bergmann-Leitner et al., 2013), while HLA-DRB1^*^04 bound to 10/11 peptides here. A previous study has shown a strong naturally-acquired humoral response in HLA-DRB1^*^04 people living in the Brazilian Amazon against 5/9 recombinant *P. vivax* proteins (Lima-Junior et al., 2012). HLA-DRB1^*^04 is one of the most frequently occurring alleles in Colombian Amerindian groups, accompanied by DR2 (DRB1^*^1602), DR6 (DRB1^*^1402) and DR8 (DRBl^*^0802). The patients' blood samples used in this study were mostly taken from Amerindians from Córdoba and Chocó. Tule (5 HLA-DRB1^*^04 alleles) is the main Amerindian population in Córdoba and the Waunana (4 HLA-DRB1^*^04 alleles) in the Chocó region, having more diverse DRBl alleles than other groups (Trachtenberg et al., 1996).

Previous studies had suggested that some alleles' over dominance in a population could be due to the spread of advantageous alleles (positive selection) after pathogen-driven selection. This has been shown by Hill et al., in a study of West African alleles (HLA-DRB1^*^1302-DQB1^*^0501) which were not present in other racial groups and were associated with protection against severe malaria, i.e., directional selection (Hill et al., 1991).

Several factors (e.g., parasite evasion mechanisms and immune molecule polymorphism) can affect protection-inducing immune responses to malarial parasites and, consequently, malaria vaccine development. Parasite evasion mechanisms include immunodominant antigen polymorphism, antigenic variation and diversion, epitope masking and the smoke-screen strategy. *P. vivax* and *P. ovale* have additional escape mechanisms which are mediated by long-lasting hypnozoites, as well as using different erythrocyte invasion pathways (Rénia and Goh, 2016). MHC Class I and II have enormous allele polymorphism and aa sequence variation in the peptide binding region, thereby enabling peptides to bind to different alleles (Blum et al., 2013). Such variability hampers a single epitope-based vaccine against *Plasmodium* parasites being developed; however, *in silico* analysis of T- and B-cell epitopes could be useful for identifying vaccine candidates represented by different epitopes which could provide coverage of the whole target population. The exposed patients studied here were grouped according to their HLA-DRB1^*^ to cover the most frequently occurring alleles in the endemic population worldwide, such as HLA-DRB1^*^04, HLA-DRB1^*^07, HLA-DRB1^*^11, and HLA- DRB1^*^13. Cell-mediated immune responses were investigated based on MHC class II peptide binding specificity and humoral immune responses by detecting antibody levels against linear B-cell epitopes.

Antibodies against *P. vivax* blood-stage proteins are important elements for blocking RBC invasion (Wipasa et al., 2002); such antibodies thus play an important role in identifying and validating *P. vivax* vaccine candidates (Soares et al., 1999; Lima-Junior et al., 2008; Storti-Melo et al., 2012; Changrob et al., 2017; Rodrigues-Da-Silva et al., 2017). We confirmed the presence of naturally-acquired humoral responses against four *Pv*RON2 B-epitopes which were recognized by IgG antibodies and subclasses. Low individual *Pv*RON2 B-epitope responder frequency was observed (20% 39041, 10% 39042, 6.6% 39043 and 39044); such low responses have previously been reported for other blood-stage antigens such as *Pv*MSP8. A loss of mean response to a target protein has been observed as time has elapsed when average response has been about ten times greater to recombinant protein than linear epitopes in acute-infection patients (Cheng et al., 2017). This has been associated with short-lived antibodies, due to short-lasting memory responses or parasite-induced B-cell dysregulation (Rénia and Goh, 2016) and parasite genetic variations or in exposed populations.

Antibody response differences against *Pv*RON2 B-cell epitopes between exposed individuals from Chocó (83%) and Córdoba (17%), could be attributed to several factors, including the level of parasitemia and the number of episodes (Druilhe and Pérignon, 1994). The last 3 SIVIGILA reports (2015–2017), showed a higher incidence of *P. vivax* infection in Chocó regarding Córdoba (Instituto-Nacional-De-Salud, 2017). Other intrinsic factors from the responders such as their HLA, sex, age, psychological stress, nutrition and other infectious diseases could also be involved in such differences (Van Loveren et al., 2001).

Two of the selected B-epitopes (39042 and 39044) were located on an α-helical coiled motif protein and other studies have shown that selected *in silico* peptides related to these motifs have been recognized by naturally-acquired antibodies and have been immunogenic in mice (Villard et al., 2007; Arévalo-Pinzón et al., 2011); however, 39042 (10%) and 39044 (6.6%) epitopes had the lowest recognition values in our study. 39041 had significant differences in the IgG subclasses analyzed; IgG2 predominated while low IgG4 and IgG1 levels were observed. A predominant IgG2 response and low IgG4 reactivity in previous studies has been associated with *P. falciparum* infection resistance and clearance, IgG2/IgG4 relationship being associated with a protective role (Aucan et al., 2000). Similar results have been found in *Pv*MSP8 studies recording IgG2 non-cytophilic antibody predominance which has been associated with resistance to *P. vivax* malaria (Cheng et al., 2017). 39041 has been seen to be immunogenic in mice (Arévalo-Pinzón et al., 2011) and its potentially protective role makes this peptide a pivotal *Pv*RON2 epitope for inclusion in a subunit-based vaccine.

It has been thought that antibodies would be enough to protect against malaria and that T-cells do not play an important role during the erythrocyte stage. Advances in immunology-related knowledge have demonstrated that B-cells must be activated by CD4+ T-helper cells to prompt good humoral responses, thereby inducing cytokine, memory cell and antibody production (Batista and Harwood, 2009; Tubo et al., 2013; Yuseff et al., 2013). The role of exposed patients' T-cell response against *Pv*RON2 high-affinity binding peptides was studied in cytokine proliferation and production assays. Only exposed individuals' PBMC cultures showed proliferation induced by universal and specific binding peptides, suggesting that *Pv*RON2 induced memory T-cells against high-affinity peptides. However, Th1 and Th2 cytokine responses were low, except for IL-6. Low cytokine responses/production have been observed in other studies; Silva-Flannery et al., found that immunization with monomeric peptide did not result in peptide-specific IFN-γ-secreting cell expansion and was not protective. They also reported that the monomeric peptide was less taken up by antigen-presenting cells and was not going through the phagolysosome (Silva-Flannery et al., 2009).

Cytokine production by unexposed individuals' PBMCs against *Pv*RON2 synthetic peptides may have been due to dendritic or macrophages cells priming naïve T-cells and inducing effector T-cell cytokine production; nevertheless, secretion was very low for some of them.

Unlike the other cytokines tested here, IL6 was highly secreted by exposed patients' PBMCs; this was not surprising, since one of IL6's multi-functions is to stimulate hybridoma and plasmacytoma cell growth and help antibody production (Matsuda et al., 1988). IL6, together with IL12 and VDR, have been associated with reduced parasitemia, its severity and gametocytemia clearance in *P. vivax*-exposed individuals (Sortica et al., 2014). *P. vivax* lysate-induced cytokine responses in unexposed individuals' PBMCs could be explained by innate immune cell cytokine production, i.e., macrophages, dendritic cells, NK, NKT and naïve T-cells which become effector cells (Stevenson and Riley, 2004). High cytokine induction in healthy individuals compared to *P. vivax*-exposed individuals might be related to a parasite evasion mechanism for inhibiting effective immune responses able to eliminate the parasite (Rénia and Goh, 2016). Nonetheless, the healthy individuals had not been exposed to *P. vivax* since immunofluorescence assays did not show their antibodies' reactivity to the parasite.

Taken together, the *in silico* T-cell and B-cell epitope selection results highlighted two T-cell epitopes (39047 and 39154) and one B-cell epitope (39041) as promising vaccine candidates. Despite the significant differences observed in immune responses evoked in the exposed individuals compared to the control group, overall the responses were relatively low. All selected peptides were conserved among the 11 *P. vivax* strains which may also explain such low immune responses. This has been demonstrated in *P. falciparum* studies where conserved high activity binding peptides (HABPs) were poorly antigenic and poorly immunogenic (Patiño et al., 1997; Lougovskoi et al., 1999; Ocampo et al., 2000; Parra et al., 2000; Hensmann et al., 2004). It should be stressed that caution must be taken, since using these 3 promising peptides in a multi-epitope vaccine in their unmodified state would probably mean that they could induce low immunogenicity and not provide long-lasting protection; however, proven approaches have shown that modifying their critical residues should induce a strong and long-lasting protection-inducing immune response (Patarroyo et al., 2010).

HABPs have been seen to be *P. falciparum* vaccine candidates during the last two decades (Rodriguez et al., 2008); however, they must be modified to make them antigenic and protection-inducing by replacing critical aa with others having the same mass but different polarity (Cifuentes et al., 2008; Patarroyo et al., 2011). Such HABPs can only be used in a tailor-made vaccine targeting a specific HLA-DRB1^*^ endemic population; however, a universal protection-inducing vaccine will require studying other peptides which can bind to other HLA-DRB1^*^ alleles. Future studies should be carried out using modified peptides aimed at assessing immunogenicity and protection-inducing ability in the *Aotus* experimental model to confirm their suitability as *P. vivax* vaccine candidates. Likewise, additional peptides should be included to cover all parasite stages, aiming at a 100% protection-inducing, multistage, multi-epitope, minimal subunit-based vaccine.

## Author contributions

CL: designed and performed the experiments, analyzed the data, drafted the manuscript. YY-P: designed and performed the experiments, analyzed the data, drafted the manuscript, designed the figures. DD-A: drafted the manuscript. MEP: critical suggestions regarding the manuscript. MAP: conceiving the work and drafting all versions of the manuscript. All authors have revised the manuscript and approved the version to be submitted.

### Conflict of interest statement

The authors declare that the research was conducted in the absence of any commercial or financial relationships that could be construed as a potential conflict of interest.

## Supplementary material

Supplementary Figure 1 Control group supernatant culture *in vitro* cytokine production. Data individual show the mean value of unstimulated and stimulated PBMC (n = 8) with universal epitope (39153), specific epitopes (39047, 39152, and 39154) and *P. vivax* lysate. IFN-γ, TNF, IL-10, and IL-6 levels were measured by CBA kit and cytokine concentration is expressed in pg/mL. Statistically significant differences (*p* ≤ 0.05) are shown and data is the means ± SEM of all values.

Supplementary Figure 2 Immunofluorescence patterns for exposed individuals from Colombia's *P. vivax*-endemic areas and the control group. The upper panels show one Bahía Solano's exposed individual serum recognition of pRBC. Nuclei were stained with DAPI, the parasite with anti-parasite FITC and then, both were merged. The bottom panels show serum from one non-exposed individual.

Supplementary Figure 3 IgG antibody response against *Pv*GAMA control recombinant protein. Significant differences (calculated by Mann-Whitney test) are shown between samples from exposed individuals and control. The dashed line indicates the cut-off point for seropositive samples.

Supplementary Table 1 Exposed-individuals' HLA-DRB1^*^ allele frequency

Supplementary Table 2 Non-exposed individuals' lymphoproliferation assay using *Pv*RON2 peptides.

## Abbreviations

aa: amino acids
AMA: apical membrane antigen
APC: antigen presenting cell
BSA: bovine serum albumin
CBA: Cytometric Bead Array
CFSE: carboxyfluorescein diacetate N-succinimidyl ester
CPD: citrate phosphate dextrose
DAPI: 4′, 6-diamidino-2-phenylinodole dihydrochloride
DNA: deoxyribonucleic acid
DBP: Duffy binding protein
ELISA: enzyme-linked immunosorbent assay
FITC: fluorescein isothiocyanate
GAMA: GPI-anchored micronemal antigen
gDNA: genomic deoxyribonucleic acid
GPI: glycophosphatidylinositol
HA: hemagglutinin antigen
HABPs: high activity binding peptides
HLA: human leucocyte antigen
HPLC: high-performance liquid chromatography
IEDB: immune epitope database
IFA: indirect immunofluorescence assays
IFN-γ: interferon gamma
IgG: immunoglobulin G
IL: interleukin
MHC: major histocompatibility complex
MJ: moving junction
Mrz: merozoites
MSP: merozoite surface protein
NGS: next generation sequencing
NK: natural killer
NKT: natural killer T-cells
OD: optical density
*P. falciparum*: *Plasmodium falciparum*
*P. vivax*: *Plasmodium vivax*
PBMC: peripheral blood mononuclear cells
PBS: phosphate buffered saline
PBST: PBS-0.05% Tween 20
pg: picograms
PHA: phytohemagglutinin
pNPP: p-nitrophenylphosphate
pRBC: parasite red blood cells
PV: parasitophorous vacuole
RALP: rhoptry-associated leucine (Leu) zipper-like protein
RBC: red blood cells
RON: rhoptry neck proteins
RPMI: roswell park memorial institute
RT: room temperature
SE: standard error
SEM: standard error of the mean
SI: stimulation index
*T. gondii*: *Toxoplasma gondii*
TBS: tris-buffered saline
TBSA: TBS-1% BSA
TCR: T-lymphocyte receptor
Th1: T helper 1
Th2: T helper 2
TMB: 3,3′,5,5′-tetramethylbenzidine
TNF: tumor necrosis factor
TRAg: tryptophan-rich antigen
TT: tetanus toxoid
VDR: vitamin D3 receptor
μM: micromolar.
