# Supplementary figures and images for: The in Vitro Antigenicity of Plasmodium vivax Rhoptry Neck Protein 2 (PvRON2) B- and T-Epitopes Selected by HLA-DRB1 Binding Profile
Source: Front Cell Infect Microbiol. 2018 May 15;8:156. doi: 10.3389/fcimb.2018.00156 (PMC5962679; doi:10.3389/fcimb.2018.00156)

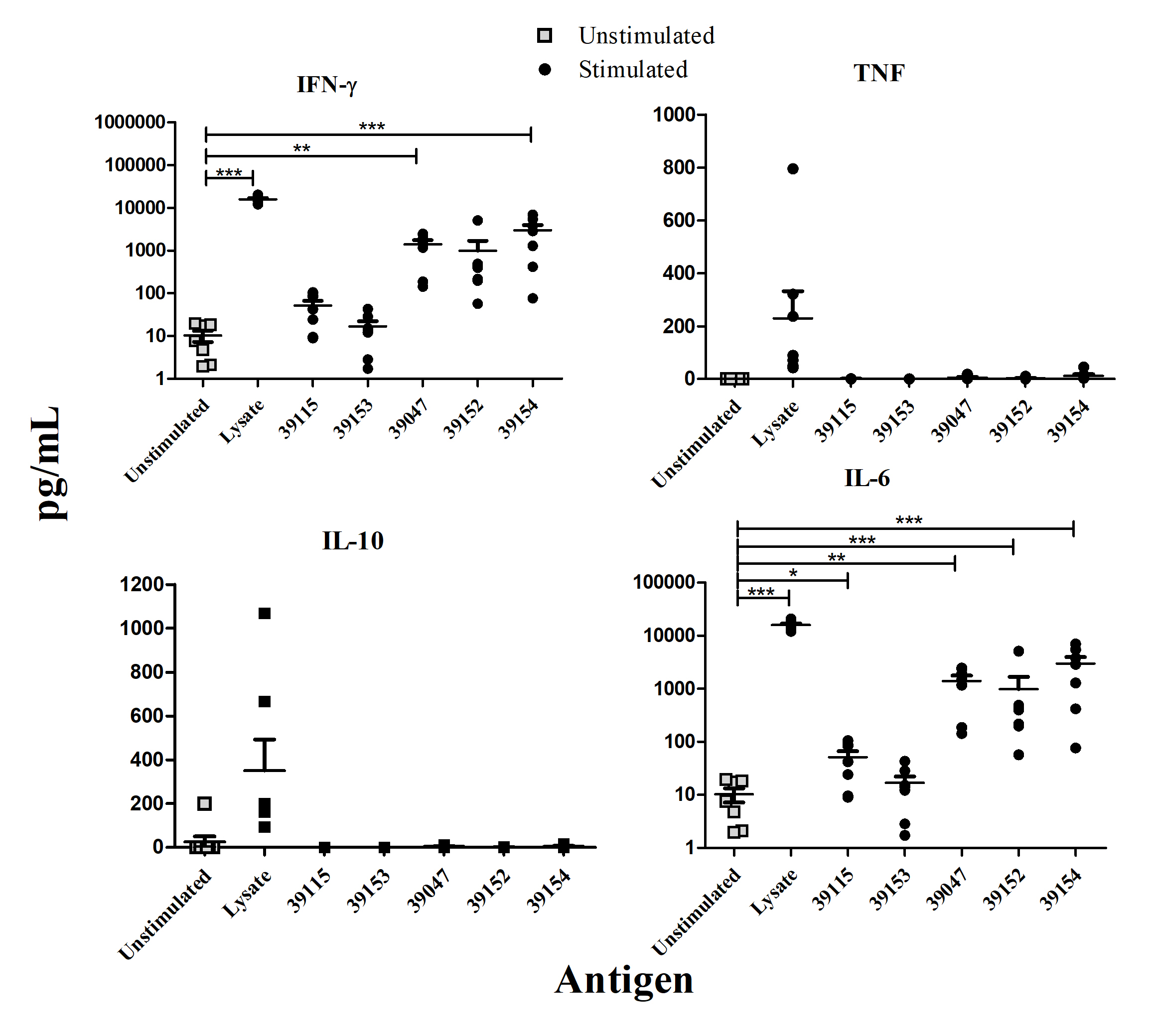

Supplement: Supplementary Figure 1 — Control group supernatant culture in vitro cytokine production. Data individual show the mean value of unstimulated and stimulated PBMC (n = 8) with universal epitope (39153), specific epitopes (39047, 39152, and 39154) and P. vivax lysate. IFN-γ, TNF, IL-10, and IL-6 levels were measured by CBA kit and cytokine concentration is expressed in pg/mL. Statistically significant differences (p ≤ 0.05) are shown and data is the means ± SEM of all values. [file Image_1.JPEG]

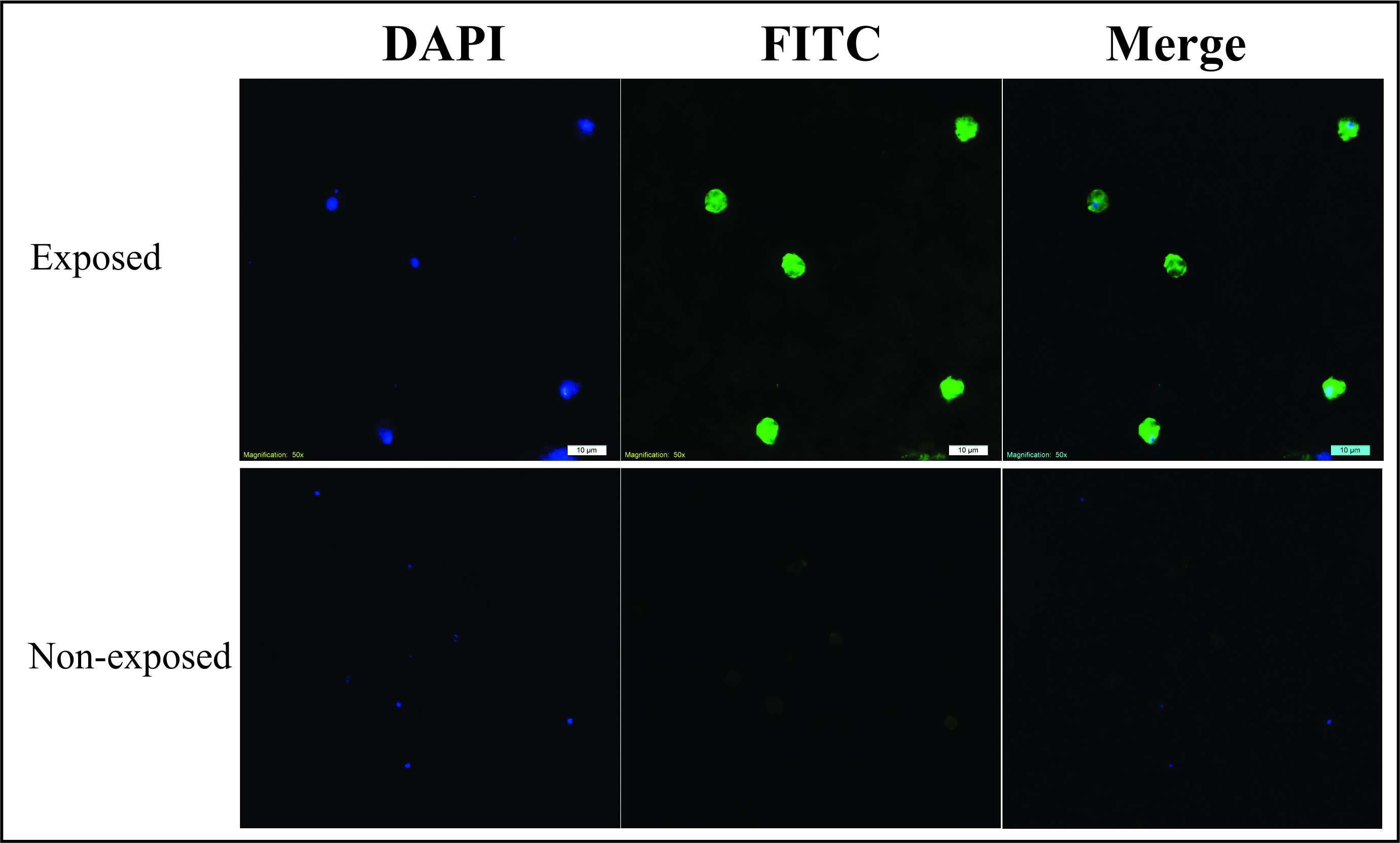

Supplement: Supplementary Figure 2 — Immunofluorescence patterns for exposed individuals from Colombia's P. vivax-endemic areas and the control group. The upper panels show one Bahía Solano's exposed individual serum recognition of pRBC. Nuclei were stained with DAPI, the parasite with anti-parasite FITC and then, both were merged. The bottom panels show serum from one non-exposed individual. [file Image_2.jpg]

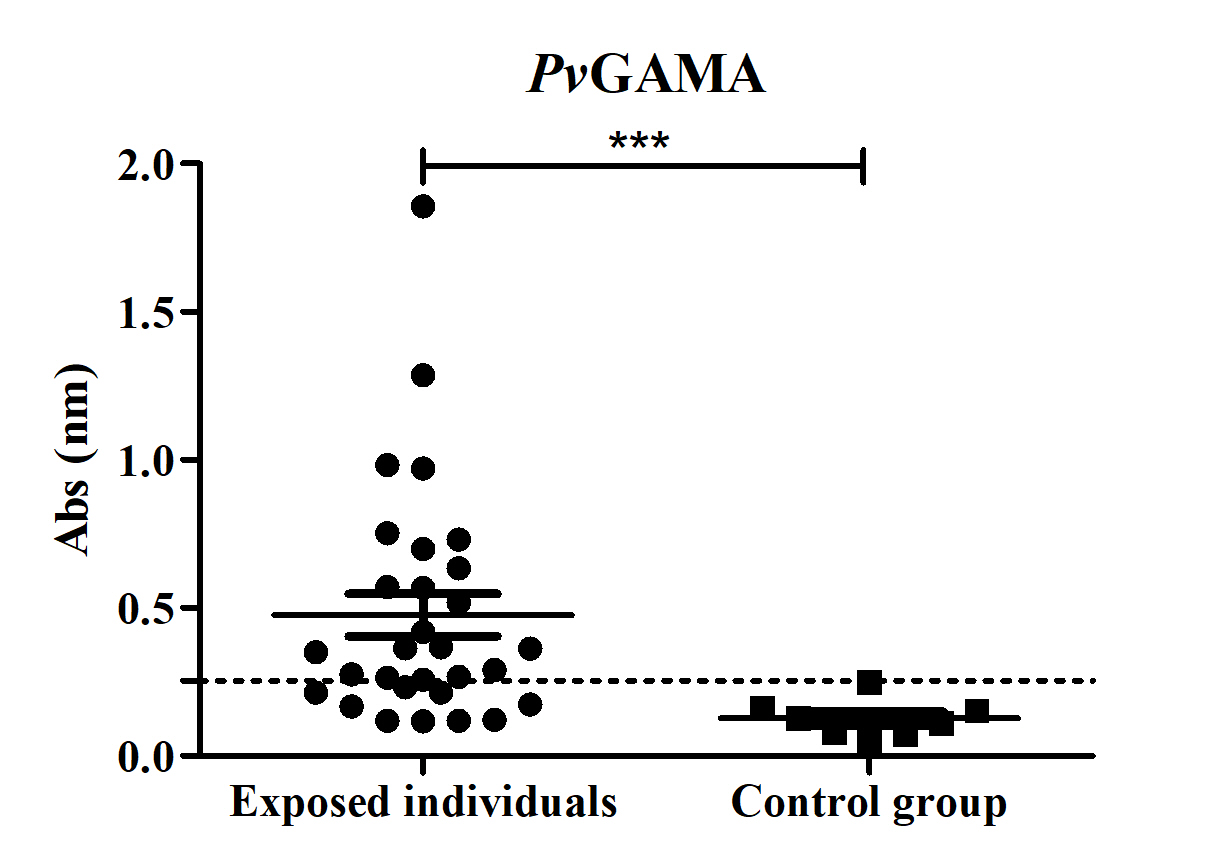

Supplement: Supplementary Figure 3 — IgG antibody response against PvGAMA control recombinant protein. Significant differences (calculated by Mann-Whitney test) are shown between samples from exposed individuals and control. The dashed line indicates the cut-off point for seropositive samples. [file Image_3.JPEG]
