# Supplementary material for: The in Vitro Antigenicity of Plasmodium vivax Rhoptry Neck Protein 2 (PvRON2) B- and T-Epitopes Selected by HLA-DRB1 Binding Profile
Source: Front Cell Infect Microbiol. 2018 May 15;8:156. doi: 10.3389/fcimb.2018.00156 (PMC5962679; doi:10.3389/fcimb.2018.00156)
Supplement: Supplementary Table 1 — Exposed-individuals' HLA-DRB1* allele frequency [file Table_1.DOCX]

**Supplementary Table 1. Exposed-individuals’ HLA DRB1* allele frequency**

| **DRB1* allele** | **n** | **Allele frequency %** |
| --- | --- | --- |
| DRB1*01 | 14 | 8.86 |
| DRB1*03 | 31 | 19.62 |
| DRB1*04 | 20 | 12.66 |
| DRB1*07 | 19 | 12.03 |
| DRB1*08 | 7 | 4.43 |
| DRB1*09 | 10 | 6.33 |
| DRB1*11 | 13 | 8.23 |
| DRB1*12 | 4 | 2.53 |
| DRB1*13 | 15 | 9.49 |
| DRB1*14 | 3 | 1.90 |
| DRB1*15 | 6 | 3.80 |
| DRB1*16 | 16 | 10.13 |
| **Total** | **158** | **100** |
