# Supplementary material for: The in Vitro Antigenicity of Plasmodium vivax Rhoptry Neck Protein 2 (PvRON2) B- and T-Epitopes Selected by HLA-DRB1 Binding Profile
Source: Front Cell Infect Microbiol. 2018 May 15;8:156. doi: 10.3389/fcimb.2018.00156 (PMC5962679; doi:10.3389/fcimb.2018.00156)
Supplement: Supplementary Table 2 — Non-exposed individuals' lymphoproliferation assay using PvRON2 peptides. [file Table_2.DOCX]

**Supplementary Table 2. Non-exposed individuals’ lymphoproliferation assay using *Pv*RON2 peptides**

|  |  | **Lymphoproliferative Response (SI)** | | | | | | |
| --- | --- | --- | --- | --- | --- | --- | --- | --- |
| **Code** | **Allele** | **Control** | **Lysate** | **39115** | **39153** | **39047** | **39152** | **39154** |
| BOG003 | DRB1*04 | 1 | 0.31 | 0.00 | 0.00 | 0.00 | 1.19 | 0.00 |
| BOG016 |  | 1 | 1.44 | 0.57 | 0.99 | 1.19 | 0.77 | 0.48 |
| BOG006 | DRB1*07 | 1 | 1.39 | 0.86 | 0.95 | 1.22 | 1.14 | 2.85 |
| BOG043 |  | 1 | 1.49 | 1.27 | 0.06 | 0.44 | 0.62 | 0.73 |
| BOG024 | DRB1*11 | 1 | 1.88 | 0.33 | 0.17 | 1.21 | 0.08 | 0.63 |
| BOG029 |  | 1 | 1.93 | 1.98 | 2.05 | 1.88 | 1.28 | 0.12 |
| BOG010 | DRB1*13 | 1 | 0.72 | 0.50 | 1.75 | 0.90 | 0.94 | 0.72 |
| BOG047 |  | 1 | 2.63 | 1.45 | 1.51 | 2.41 | 2.01 | 1.58 |
